# Rhynchophylline Protects Against Ischemic Injury Following Myocardial Infarction via Activation of the SIRT1/NRF2/FOXO3a Axis

**DOI:** 10.3390/antiox15060669

**Published:** 2026-05-26

**Authors:** Wenyue Yang, En Ma, Zihan Zhou, Lingyun Yang, Jinxiao Chen, Weidong Zhu, Dan-ni Ren, Da Wo

**Affiliations:** Academy of Integrative Medicine, College of Integrative Medicine, Fujian Key Laboratory of Integrative Medicine on Geriatric, Fujian University of Traditional Chinese Medicine, Fuzhou 350122, China; 13253011860@163.com (W.Y.); 18217497624@163.com (E.M.); 13679039069@163.com (Z.Z.); y19862177149@163.com (L.Y.); shangtang002002@163.com (J.C.); wzhu@tongji.edu.cn (W.Z.)

**Keywords:** rhynchophylline, myocardial infarction, SIRT1/NRF2/FOXO3a, oxidative stress, ischemia

## Abstract

Myocardial infarction (MI) remains the leading cause of death globally. Current treatment strategies involve restoring blood flow to the coronary artery, but have shortcomings in that these procedures cannot reverse damage to the myocardium that has already occurred. Therefore, therapies that can decrease the severity of ischemic damage are needed. Oxidative stress is an early and major driver of cardiomyocyte death following MI. Rhynchophylline (RHY) is a natural alkaloid known for its antioxidant activity; however, whether it can protect against MI-induced ischemic injury, as well as its underlying mechanism of action, remains unexplored. We performed murine models of surgical MI and examined the effects and mechanisms of RHY in protecting against myocardial ischemic injury. A sirtuin 1 (SIRT1)-specific inhibitor, EX-527, was subsequently used to verify that the cardioprotective effects of RHY were dependent upon targeted SIRT1-activation. Mice administered with RHY significantly protected against ischemic injury following MI, with improved cardiac function, reduced infarct size, and decreased levels of oxidative and DNA damage. The cardioprotective effect of RHY is associated with activation of the SIRT1 and its downstream redox-sensitive transcription factors: nuclear factor erythroid 2-related factor 2 (NRF2) and forkhead-box protein O3 (FOXO3a). The cardioprotective and antioxidant effects of RHY were abolished by EX-527, a selective SIRT1 inhibitor. Our findings provide evidence for the robust antioxidant properties of RHY in protecting against MI injury via activating the SIRT1/NRF2/FOXO3a signaling axis. These findings provide new mechanistic insight into the preconditioning-like cardioprotective potential of RHY during myocardial infarction.

## 1. Introduction

Ischemic heart disease (IHD) remains the leading cause of mortality worldwide [1], among which acute myocardial infarction (MI) is the most common pathological mechanism. Current clinical treatments of MI include thrombolysis, percutaneous coronary intervention (PCI), and coronary artery bypass grafting [2], but these approaches cannot reverse damage to the ischemic myocardium due to the lack of cardiomyocyte regenerative potential [3,4]. The early onset of MI is characterized by excessive production of reactive oxygen species (ROS), leading to mitochondrial dysfunction, DNA damage, and cardiomyocyte death. Therefore, decreasing the severity of myocardial ischemic damage following MI may be just as important for improving patient prognosis [5].

In order to eliminate ROS, the body’s endogenous antioxidant defense network acts by activating numerous redox pathways and restoring intracellular redox homeostasis. One of the most important pathways is the activation of the highly conserved sirtuin family of protein deacetylases, including Sirtuin 1 (SIRT1), in response to cellular stress and changes in intracellular metabolism. SIRT1 can directly deacetylate and promote the function of multiple transcription factors or co-factors, including the forkhead-box family of transcription factors (FOXO) and nuclear factor erythroid 2-related factor 2 (NRF2) [6,7]. Studies have shown that drugs with SIRT1-targeted activation exhibited beneficial effects in numerous ischemic heart disease models [8,9,10,11,12], by enhancing ROS scavenging capacity, maintaining mitochondrial homeostasis, and inhibiting apoptosis. Recent studies have highlighted a synergistic interaction between SIRT1 and the redox-sensitive NRF2 and FOXO3a transcription factors [13], both of which are critical in regulating the genes responsible for the oxidative stress response. A major contributor to the exacerbation of ischemic injury is a lack of intrinsic NRF2- or FOXO3a-regulated intracellular protective response, which is often suppressed during MI. Therefore, discovering safe and effective drugs that can target the activation of SIRT1 and its downstream effectors during extreme oxidative stress, such as MI, presents a promising preconditioning-like cardioprotective potential against ischemic injury.

Natural active compounds extracted from plants have attracted increasing interest as potential novel drugs in various diseases due to their relatively favorable safety profiles and multi-target properties. Recent studies have shown that rhynchophylline (RHY), an indole alkaloid extracted from Uncaria rhynchophylla, exhibits various anti-inflammatory, antioxidative, and neuroprotective effects during cerebral ischemia [14], neurodegeneration [15], and inflammatory disorders [16]. Although several studies have suggested potential cardiovascular benefits of RHY in preventing cardiac dysfunction in non-ischemic settings [17,18], no study has yet examined whether RHY can also exhibit any cardioprotective effects during acute MI, and its underlying mechanisms of action remain unexplored. Preliminary experiments demonstrated strong antioxidative and SIRT1 activation ability of RHY, and hence we hypothesized that RHY may protect the heart during myocardial infarction by activating the SIRT1/NRF2/FOXO3a signaling axis and guarding against MI-induced oxidative stress and DNA damage. Our current study thus aimed to elucidate the cardioprotective effects and mechanisms of RHY, in particular its potential preconditioning-like cardioprotective effect during MI.

## 2. Materials and Methods

### 2.1. Drugs and Reagents

RHY (purity 99.75%) was purchased from MedChemExpress (MCE, Cat. No. 76-66-4, Shanghai, China). For in vivo experiments, a 40 mg/mL stock solution was prepared in dimethyl sulfoxide (DMSO) and stored at −80 °C until use. Before administration, the stock solution was diluted with phosphate-buffered saline (PBS) to the appropriate working concentrations. Mice received administrations of RHY at either low dose (20 mg/kg/day) or high dose (40 mg/kg/day), while the control group received an equal volume of vehicle solution.

For in vitro experiments, RHY was dissolved in DMSO to prepare an 80 mM stock solution. The stock solution was diluted with culture medium to obtain final concentrations of 5, 10, 20, and 40 μM. Cells in the control group were treated with an equivalent volume of vehicle solution.

### 2.2. Animal Studies

All animal procedures were performed in accordance with the Guide for the Care and Use of Laboratory Animals published by the National Institutes of Health and followed the ARRIVE guidelines. The study protocol was approved by the Experimental Animal Care and Use Committee of Fujian University of Traditional Chinese Medicine. (Approval No.: FJTCM IACUC 2025135, Approval date: 29 May 2025).

Male C57BL/6J mice (8 weeks old, 25–28 g) were purchased from Shanghai SLAC Laboratory Animal Co., Ltd. (Shanghai, China). Mice were acclimatized for at least one week under standard specific pathogen-free (SPF) conditions (24 ± 1 °C, 12 h light/dark cycle) with free access to food and water prior to surgery. Mice were first weighed then stratified according to body weight by a blinded investigator to ensure comparable baseline body weights across all experimental groups as follows: (1) sham-operated control (Sham), (2) MI model (MI + PBS), (3) RHY low dose (MI + 20 mg/kg RHY), (4) RHY high dose (MI + 40 mg/kg RHY), with at least 8 mice allocated to each group. Mice received daily administration of RHY via intraperitoneal injection, starting three days prior to surgery until four weeks post-surgery. Sham and MI model groups received an equal volume of vehicle solution.

The myocardial infarction (MI) model was established by permanent ligation of the left anterior descending coronary artery (LAD), which was consistent with previous studies [19,20]. All mice were equally healthy with similar body weight, and hence there were no exclusions. Briefly, mice were anesthetized and intubated with a ventilator. The chest cavity was held open using a retractor, the pericardium was gently separated, and the LAD was permanently ligated using a 7-0 silk suture. Sham-operated mice underwent the same procedure, but the suture was passed under the LAD without ligation. There were no failed ligations as confirmed by echocardiography assessment. The mortality data post-surgery were as follows: no deaths in the SHAM group, 4 deaths in the MI + PBS group at day-0, day-1, day-4, and day-15 respectively, post-MI, 1 death in the MI + RHY 20 mg/kg group at day-6 post-MI, 3 deaths in the MI + RHY 40 mg/kg group at day-0, day-1, and day-3 post-MI. After 4 weeks, mice were euthanized via overdose of sodium pentobarbital, and blood and heart tissues were immediately collected for subsequent histological and molecular analyses. All animal surgical procedures and subsequent analyses were performed by a blinded investigator.

### 2.3. Two-Dimensional Echocardiography

Two-dimensional echocardiography was performed using the VisualSonics Vevo 2100 Imaging System. Mice were anesthetized with 1% isoflurane (Sigma-Aldrich, St. Louis, MO, USA) in oxygen delivered via a vaporizer (EZ Anesthesia, Palmer, PA, USA). Heart rate was monitored and maintained at 400–450 beats per minute throughout the procedure. M-mode images were acquired to assess left ventricular (LV) dimensions, including left ventricular internal diameter at end-diastole (LVID;d) and end-systole (LVID;s), as well as left ventricular posterior wall thickness at end-diastole (LVPW;d). LV volumes at end-diastole (LV Vol;d) and end-systole (LV Vol;s) were calculated from LV dimensions. Stroke volume (SV) was determined as the difference between LV Vol; d and LV Vol; s. LV ejection fraction (EF%) was calculated as (LV Vol;d−LV Vol;s)/LV Vol; d ×100 and fractional shortening (FS%) was calculated as (LVID;d−LVID;s)/LVID; d × 100.

### 2.4. Histological Analysis

Mouse hearts were fixed in 4% paraformaldehyde for 24 h, then washed overnight in running water, followed by dehydration through a graded ethanol series, xylene clearance, and paraffin embedding. Sections were sliced to 5 µm thickness and stained with Masson’s trichrome staining, where collagen deposits were stained in blue and viable muscle fibers were stained in red. Images were captured under a Leica Aperio VERSA optical microscope at 20× magnification, and quantitative analysis was performed using Image-Pro Plus 6.0. The infarct size was calculated as the ratio of infarcted endocardial circumference/total endocardial circumference × 100%.

### 2.5. Immunofluorescence Staining

Fresh heart tissues were embedded in Tissue-Tek OCT compound, rapidly frozen on dry ice, and sectioned to 5 µm thickness on positively charged hydrophilic slides. Sections were washed with PBS, fixed with 4% paraformaldehyde for 15 min, permeabilized with 0.25% Triton X-100, blocked with 5% BSA for 1 h and finally incubated overnight at 4 °C with the following primary antibodies: Actn2 (Sigma Aldritch, St. Louis, Missouri, USA, Cat. No. A7811) as a cardiomyocyte-specific reference stain, together with FOXO3a (Abcam, Cat. No. ab23683, Street Waltham, MA, USA), NRF2 (Proteintech, Cat. No. 16396-1-AP, Rosemont, IL, USA), γ-H2AX (Abcam, Cat. No. ab81299), or 3-NT (Abcam, Cat. No. ab110282). Sections were then incubated with Alexa Fluor 488-conjugated anti-mouse (Abcam, Cat. No. ab150113) and Alexa Fluor 647-conjugated anti-rabbit secondary antibodies (Abcam, Cat. No. ab150115) for 1 h and mounted with anti-fade medium containing DAPI (Beyotime, Cat. No. P0131, Shanghai, China). Immunofluorescence images were acquired using a Leica Stellaris 8 Fa confocal microscope at 20× magnification. Fluorescence signals were analyzed with ImageJ software (version 1.53s), while nuclear-localized foci were counted if >50% of positive staining was localized in the nucleus.

### 2.6. Hydrogen Peroxide Colorimetric Assay

Hydrogen peroxide levels were measured using a hydrogen peroxide colorimetric assay kit (Abcam, Cat. No. ab102500) according to the manufacturer’s instructions. Briefly, equal amounts of mouse heart tissue were thoroughly homogenized on ice and deproteinized with perchloric acid, followed by pH neutralization using potassium hydroxide. Samples and standards were added to a 96-well plate, incubated for 10 min in the dark, and the absorbance was measured at 570 nm. The final hydrogen peroxide concentration of tissues was calculated based on the standard curve.

### 2.7. Cell Culture

In vitro experiments were conducted using H9c2 cardiomyocytes. The H9c2 rat embryonic cardiomyoblast cell line was obtained from the American Type Culture Collection (ATCC, Manassas, VA, USA). All experiments were performed using cells at passage 3 to minimize variability associated with prolonged culture. Cells were cultured in high-glucose DMEM supplemented with 10% fetal bovine serum (FBS) and 1% penicillin–streptomycin at 37 °C in 5% CO_2_. Cells were treated under the following conditions: RHY alone (5–80 μM, 12 h), RHY + H_2_O_2_ (200 μM, 1 h), SIRT1 inhibitor alone (EX-527, 1–40 μM, 12 h). Experiments were performed at least three times.

### 2.8. Cell Viability Assay (CCK-8)

Cell viability was assessed using the CCK-8 assay (Beyotime, Shanghai, China). Briefly, H9c2 cells were seeded in 96-well plates and treated with various concentrations of RHY for 12 h. Subsequently, cell culture medium was replaced with CCK-8 reagent (1:10 dilution) in fresh medium. After incubation for 2 h at 37 °C in the dark, absorbance was measured at 450 nm using a microplate reader (Thermo Fisher, Waltham, MA, USA). Relative cell viability was calculated relative to the control.

### 2.9. Western Blotting

Western blot analyses were performed following standard protocols. Total protein and nuclear protein were extracted using Protein Extraction Kit (Beyotime, Cat. No. P0013F) and Nuclear Protein Extraction Kit (Sangon Biotech, Cat. No. C500009, Shanghai, China), respectively, according to the manufacturers’ instructions. Protein concentrations were determined using the BCA assay, and equal amounts of protein were separated by SDS-PAGE and transferred onto 0.22 μm PVDF membranes. Membranes were blocked with 5% non-fat milk and incubated overnight at 4 °C with the following primary antibodies: TBP (CST, Cat. No. 8515, Danvers, MA, USA), β-actin (CST, Cat. No. 4970s), Alpha actin (Proteintech, Cat. No. 66125-1-Ig, Rosemont, IL, USA), ANP (Abcam, Cat. No. ab225844), BNP (Abcam, Cat. No. ab236101), cTnI (Abcam, Cat. No. ab47003), SIRT1 (Proteintech, Cat. No. 13161-1-AP), NRF2 (Proteintech, Cat. No. 16396-1-AP), FOXO3a (Abcam, Cat. No. ab23683), Acetyl-FOXO3a (Lys271, Affinity Biosciences, Changzhou, China, Cat. No. #AF3771), and Acetyl-NRF2 (Lys599, Immunoway, Cat. No. YK0063, San Jose, CA, USA). Membranes were then incubated with the corresponding HRP-conjugated secondary antibodies, and protein bands were visualized via chemiluminescence.

### 2.10. Statistical Analysis

All statistical analyses were performed using GraphPad Prism 9.5.0. Data were normalized to the control or SHAM group and presented as relative values. Normality and homogeneity of variance were assessed prior to analysis. Comparisons between two groups were conducted using an unpaired Student’s *t*-test. For multiple group comparisons at one data point, we used one-way ANOVA followed by Fisher’s LSD post hoc test. For analysis derived from two data points, we used a mixed-effects model with Sidak’s multiple comparisons test. A *p* < 0.05 was considered statistically significant.

## 3. Results

### 3.1. RHY Improves Cardiac Function Following Myocardial Infarction (MI)

**Figure 1:**
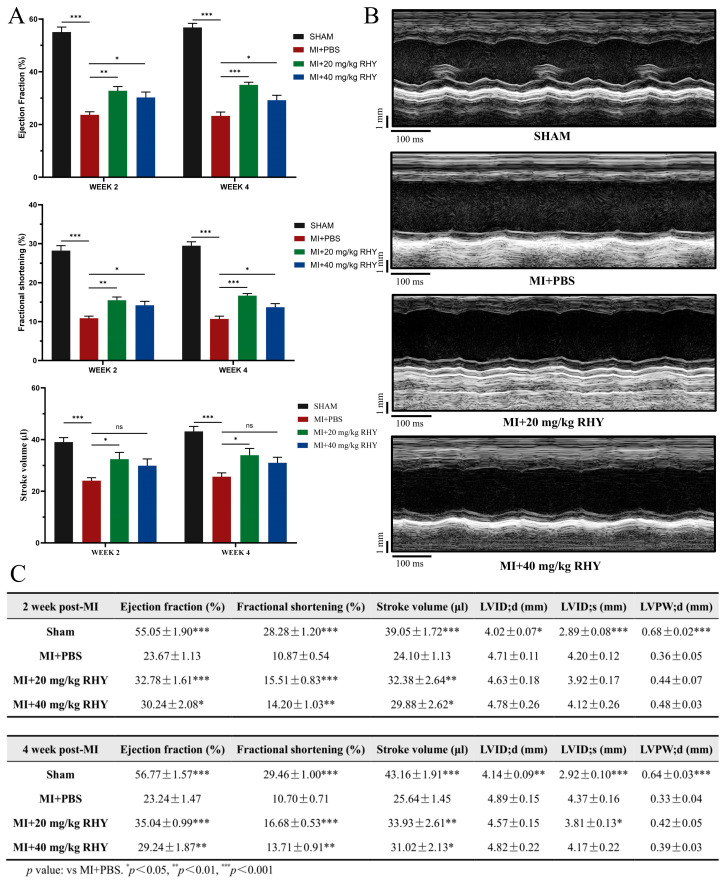
RHY improves cardiac function in mice following myocardial infarction (MI). (**A**) Cardiac function parameters, including ejection fraction (EF%), fractional shortening (FS%), and stroke volume (SV), in mice administered with PBS, low-dose RHY (20 mg/kg/day), or high-dose RHY (40 mg/kg/day) at 2 weeks and 4 weeks post-MI. (**B**) Representative M-mode echocardiography at 4 weeks post-MI showing LV end-systolic and end-diastolic dimensions in PBS-treated or RHY-treated mice. (**C**) Table of cardiac function parameters in Sham, MI, low-dose RHY (20 mg/kg/day), or high-dose RHY (40 mg/kg/day) at 2 weeks and 4 weeks post-MI, including left ventricular internal diameters at end-diastole (LVID;d) and end-systole (LVID;s), and left ventricular posterior wall thickness at end-diastole (LVPW;D). Data are presented as mean ± SEM, mixed-effects model with Sidak’s multiple comparisons test. Adjusted *p* values shown * *p* < 0.05, ** *p* < 0.01, *** *p* < 0.001, ns, no significance. SHAM: *n* = 7; MI + PBS: *n* = 11; MI + 20 mg/kg RHY: *n* = 7; MI + 40 mg/kg RHY: *n* = 10.

We first investigated the preconditioning-like cardioprotective potential of rhynchophylline (RHY) following MI via permanent ligation of the left anterior descending coronary artery in mice. Echocardiographic assessment at 2- and 4 weeks post-MI showed that RHY administration improved cardiac function post-MI, as evidenced by key indicators of cardiac function, including left ventricular ejection fraction (EF), fractional shortening (FS), and stroke volume (SV), which were markedly rescued in mice administered with RHY at both low- and high-dosages (Figure 1A). Consistent with this finding, echocardiograms showed a significant improvement in left ventricular wall motion in RHY-administered mice post-MI compared to PBS-administered mice (Figure 1B). The summary of echocardiographic parameters was summarized in (Figure 1C), including LV wall thickness, which also saw improving trends following RHY administration post-MI but without statistical significance. Mice administered with high-dose RHY had similar, but no increased benefits compared to a low-dose, suggesting that low-dose RHY is already enough to warrant cardioprotection post-MI. These results collectively demonstrate a beneficial cardioprotective effect of RHY on cardiac function following MI.

### 3.2. RHY Decreases the Extent of Myocardial Injury and Remodeling Following MI

**Figure 2:**
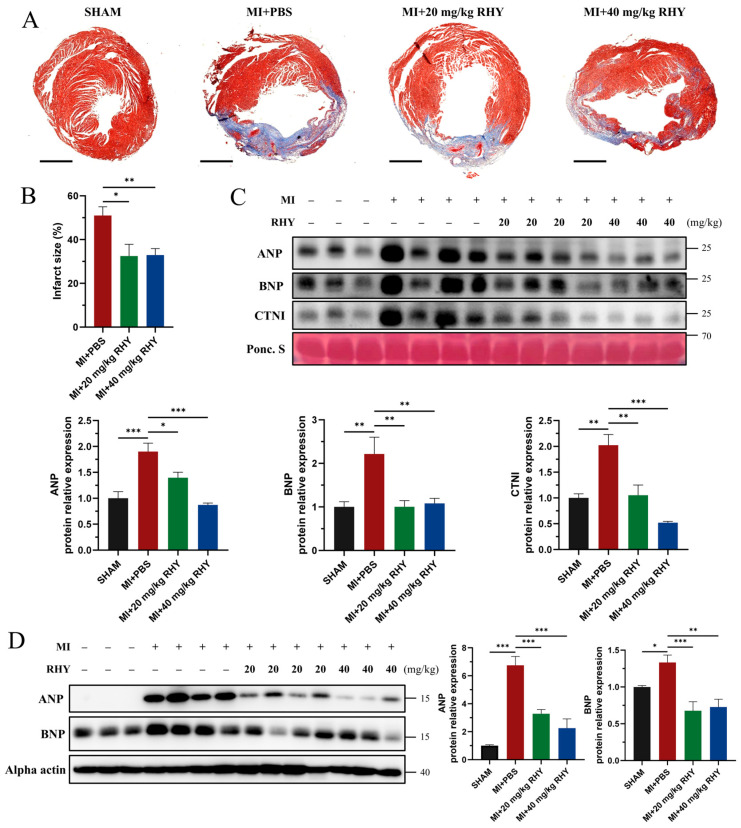
RHY attenuates myocardial injury and remodeling. (**A**,**B**) Representative Masson’s trichrome-stained heart tissue sections (**A**) and quantification of intraventricular infarct size based on collagen scarring (**B**) in heart tissue at 4 weeks post-MI in PBS- and RHY-treated mice. Muscle fibers are stained red, and collagen-rich fibrotic regions are stained blue. 20× objective lens. Scale bar = 1 mm. For histology: SHAM: *n* = 3; MI + PBS: *n* = 4; MI + 20 mg/kg RHY: *n* = 5; MI + 40 mg/kg RHY: *n* = 5. (**C**,**D**) Representative immunoblot and quantification of atrial natriuretic peptide (ANP), B-type natriuretic peptide (BNP), and cardiac troponin I (cTnI) expression in the serum (**C**) and cardiac tissue (**D**) of mice at 4 weeks post-MI. Data are presented as mean ± SEM. One-way ANOVA, * *p* < 0.05, ** *p* < 0.01, *** *p* < 0.001. For serum markers: SHAM: *n* = 3; MI + PBS: *n* = 4; MI + 20 mg/kg RHY: *n* = 4; MI + 40 mg/kg RHY: *n* = 3.

Next, we evaluated the impact of RHY on myocardial injury and pathological remodeling following MI. Masson’s trichrome staining of cardiac tissues at 4 weeks post-MI showed a significant degree of myocardial fibrotic scar formation that was reflected in the 50% cardiac infarct size based on endocardial circumference (Figure 2A,B). Notably, mice administered with RHY at both dosages had significantly reduced infarct sizes compared to the PBS-administered mice (Figure 2B). Moreover, key markers of myocardial injury, including atrial natriuretic peptide (ANP), brain natriuretic peptide (BNP), and cardiac troponin I (cTnI) [21], were significantly elevated following myocardial infarction in both the serum (Figure 2C) and heart lysates (Figure 2D). Although cTnI is classically considered a marker of acute myocardial injury, its persistence at 4 weeks post-MI might reflect ongoing myocardial damage/remodeling rather than acute troponin release associated with acute cardiomyocyte necrosis. Importantly, RHY-administered mice at both dosages markedly prevented the elevations of these myocardial injury biomarkers (Figure 2C,D). These results collectively demonstrate that RHY administration prevents pathological remodeling of the heart and decreases the extent of myocardial injury following MI.

### 3.3. RHY Decreases the Degree of Oxidative and DNA Damage in the Ischemic Heart

**Figure 3:**
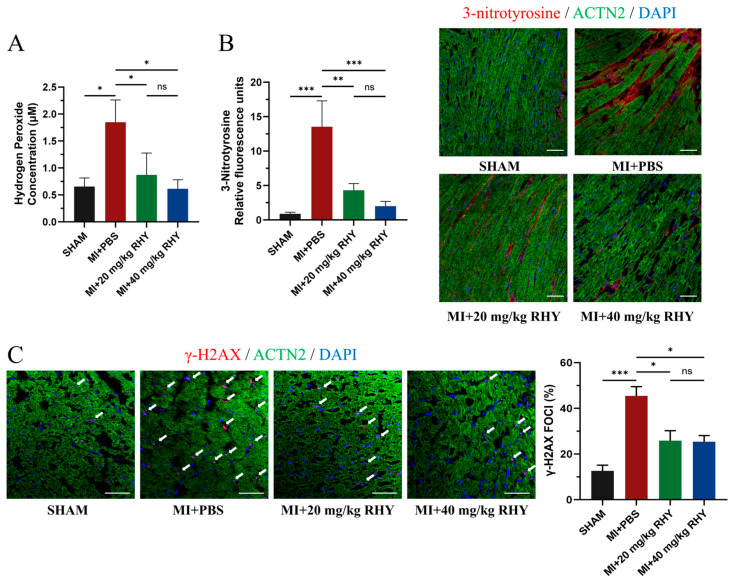
RHY reduces oxidative stress and DNA damage in infarcted hearts. (**A**) Statistical analysis of hydrogen peroxide (H_2_O_2_) levels in heart tissue at 4 weeks post-MI, measured using a hydrogen peroxide colorimetric assay. For hydrogen peroxide assay: SHAM: *n* = 5; MI + PBS: *n* = 6; MI + 20 mg/kg RHY: *n* = 6; MI + 40 mg/kg RHY: *n* = 5. (**B**) Representative immunofluorescence images (right) showing 3-nitrotyrosine (3-NT, red), ACTN2 (green), and DAPI (blue) in heart tissue at 4 weeks post-MI. 20× objective lens. Scale bar = 50 µm. Quantification (left) of 3NT fluorescence intensity in PBS- and RHY-treated mice myocardial cells, normalized to the SHAM group, showing relative fluorescence intensity. (**C**) Representative immunofluorescence images (left) showing phosphorylated histone H2AX foci (γ-H2AX, red), cardiac muscle marker alpha-actinin-2 (ACTN2, green), and DAPI (blue) in heart tissue at 4 weeks post-MI. White arrows denote nuclear γ-H2AX-positive foci. 20× objective lens. Scale bar = 50 µm. Quantification (right) of γ-H2AX-positive cells in PBS- and RHY-treated mice myocardial cells in the ischemic heart. Data are presented as mean ± SEM. One-way ANOVA, * *p* < 0.05, ** *p* < 0.01, *** *p* < 0.001, ns, no significance. For IF analysis: SHAM: *n* = 6; MI + PBS: *n* = 7; MI + 20 mg/kg RHY: *n* = 7; MI + 40 mg/kg RHY: *n* = 6; for each animal, two separate sections with at least four fields were used for quantification.

Myocardial infarction results in the excessive generation of reactive oxygen species (ROS) that results in DNA damage and cardiomyocyte death in the infarcted myocardium [22]. RHY-induced improvements in cardiac function post-MI can be translated to a decreased degree of oxidative and DNA damage in the ischemic heart. Hence, we examined the levels of endogenous hydrogen peroxide (H_2_O_2_) in the cardiac tissues post-MI via colorimetric assay to determine the extent of ROS accumulation. Mice that underwent MI had significantly increased levels of H_2_O_2_ at 4 weeks post-MI compared to sham-operated mice, while RHY-administered mice had significantly decreased levels of endogenous H_2_O_2_ at both dosages (Figure 3A). Next, we examined the levels of endogenous nitrogen-free radicals in the ischemic myocardium by detecting 3-nitrotyrosine (3-NT), a product of protein tyrosine nitration [23]. Immunofluorescence staining revealed extensive generation of 3-NT in the border zone of ischemic myocardium in PBS-administered mice that underwent MI, whereas RHY administration markedly decreased the extent of 3-NT formation post-MI, most obviously in the high-dose RHY group (Figure 3B). Moreover, we examined the level of DNA damage in the ischemic myocardium as a result of MI by detecting phosphorylated histone H2AX (γ-H2AX), an early and sensitive marker of DNA double-strand breaks. Immunofluorescence staining showed a significant increase in the number of γ-H2AX positive foci in the border zone of ischemic myocardium of mice post-MI compared to sham-operated mice, which was markedly reduced in mice administered with RHY (Figure 3C). Collectively, these findings demonstrate the beneficial effect of RHY in decreasing the extent of oxidative stress and DNA damage occurrence following myocardial infarction.

### 3.4. RHY Activates the SIRT1–NRF2/FOXO3a Signaling Axis In Vivo

**Figure 4:**
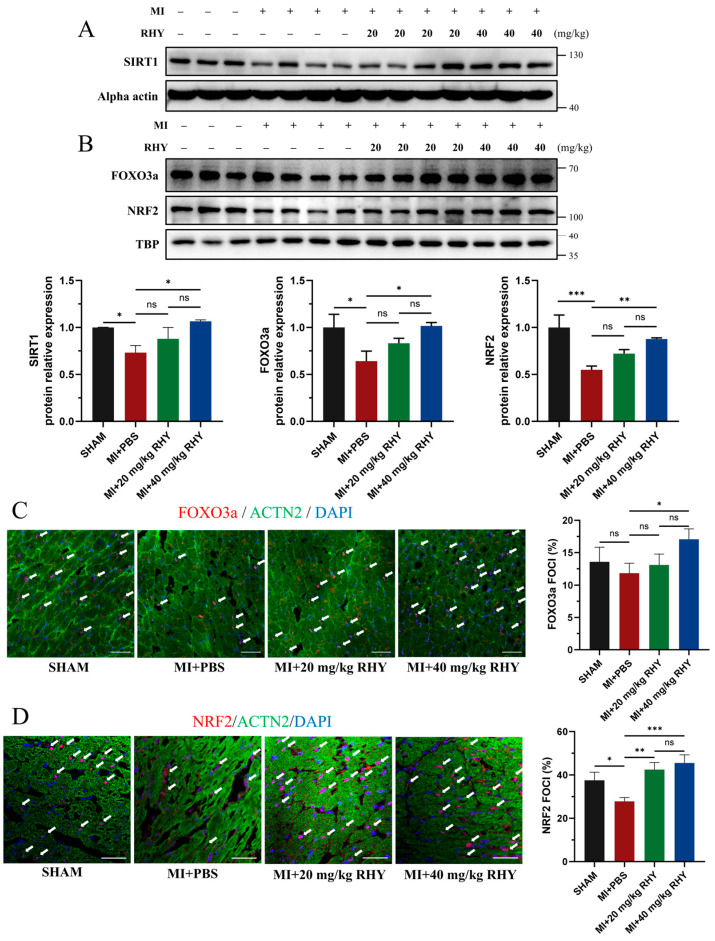
RHY provides cardioprotection following myocardial infarction via activating the SIRT1/NRF2/FOXO3a axis. (**A**,**B**) Representative immunoblots (top left) showing the expression of SIRT1 in total protein (**A**) and FOXO3a and NRF2 in nuclear protein (**B**) from heart tissue at 4 weeks post-MI. Quantification of gray values normalized to internal controls, alpha actin for total protein (**A**) and TBP for nuclear protein (**B**). For WB analysis: SHAM: *n* = 3; MI + PBS: *n* = 4; MI + 20 mg/kg RHY: *n* = 4; MI + 40 mg/kg RHY: *n* = 3. (**C**) Representative immunofluorescence images (left) showing FOXO3a expression (red), ACTN2 (green), and DAPI (blue) in heart tissue at 4 weeks post-MI. 20× objective lens. Scale bar = 50 µm. Quantification (right) of nuclear FOXO3a-positive cells, with statistical analysis of the nuclear entry rate. (**D**) Representative immunofluorescence images (left) showing NRF2 expression (red), ACTN2 (green), and DAPI (blue) in heart tissue at 4 weeks post-MI. 20× objective lens. Scale bar = 50 µm. Quantification (right) of nuclear NRF2-positive cells, with statistical analysis of the nuclear entry rate. White arrows denote nuclear FOXO3a- or NRF2-positive foci. For IF analysis: SHAM: *n* = 6; MI + PBS: *n* = 7; MI + 20 mg/kg RHY: *n* = 7; MI + 40 mg/kg RHY: *n* = 6; for each animal, two separate sections with at least four fields were used for quantification. Data are presented as mean ± SEM. One-way ANOVA, * *p* < 0.05, ** *p* < 0.01, *** *p* < 0.001, ns, no significance.

Sirtuin 1 (SIRT1) is a key regulator of redox homeostasis and plays critical roles in MI by preventing oxidative stress in the ischemic myocardium caused by excessive ROS production. Hence, we investigated whether RHY-induced improvement in cardiac function post-MI and protection against oxidative stress was via activating SIRT1 and its downstream transcription factors involved in the elimination of ROS. At four weeks post-MI, the total protein level of SIRT1 was significantly decreased compared to sham-operated mice, suggesting that the intrinsic SIRT1-mediated response is naturally suppressed following MI (Figure 4A). Supporting this notion, the nuclear protein levels of downstream redox-sensitive transcription factors FOXO3a and NRF2 were also significantly decreased following MI (Figure 4B), indicative of impaired intracellular protective response against oxidative stress. Notably, mice administered with RHY markedly restored both the levels of SIRT1 and NRF2/FOXO3a, especially in the high-dose group, exhibiting a clear gradient of effect (Figure 4A,B). Moreover, RHY-administered mice exhibited significantly decreased levels of acetylated-FOXO3a and acetylated-NRF2, further demonstrating that RHY activates SIRT1 and deacetylates NRF2/FOXO3a, thereby promoting their transcriptional activity (Supplemental Figure S1). Taken together, these results demonstrate that RHY can target the SIRT1-mediated antioxidant network and activate intrinsic NRF2- and FOXO3a-regulated response to oxidative stress.

We further performed immunofluorescence staining to validate the functional activation of FOXO3a and NRF2 transcription factors in mice following RHY administration. The number of NRF2-positive nuclear foci was significantly decreased following MI induction compared to sham-operated mice. However, although there was no significant reduction in FOXO3a-positive nuclear foci, RHY-administered mice significantly increased the numbers of FOXO3a and NRF2-positive foci in the border zone of ischemic myocardium, most notably FOXO3a, where there was a clear gradient of effect for high-dose RHY (Figure 4C,D). These results demonstrate that RHY administration promotes the activation of key redox-sensitive transcription factors following ischemic injury and that the cardioprotective effect of RHY in MI was via activation of the SIRT1/NRF2/FOXO3a signaling axis.

### 3.5. SIRT1 Inhibition Abolishes the Cardioprotective Effects of RHY

**Figure 5:**
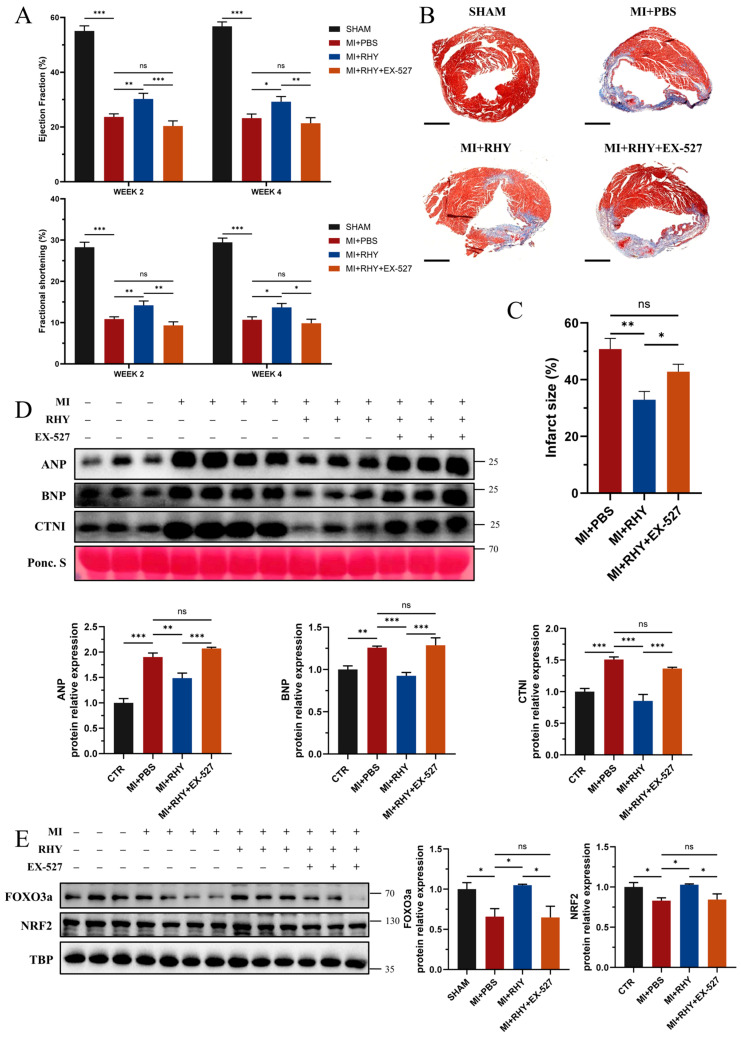
Administration of selective SIRT1 inhibitor (EX-527) abolishes the cardioprotective effects of RHY following myocardial infarction. (**A**) Cardiac function parameters, including ejection fraction (EF%) and fractional shortening (FS%), in mice treated with RHY (40 mg/kg) and/or SIRT1 inhibitor (EX-527, 10 mg/kg/day) at 2 weeks and 4 weeks post-MI. Mixed-effects model with Sidak’s multiple comparisons test. Adjusted *p* values shown *p < 0.05, **p < 0.01, ***p < 0.001. For echocardiography: SHAM: *n* = 7; MI + PBS: *n* = 11; MI + RHY: *n* = 10; MI + RHY + EX-527: *n* = 5. (**B**,**C**) Representative Masson’s trichrome-stained heart tissue sections (**B**) and quantification (**C**) of intraventricular infarct size based on collagen scarring in heart tissue at 4 weeks post-MI in mice treated with EX-527. Muscle fibers are stained red, and collagen-rich fibrotic regions are stained blue. 20× objective lens. Scale bar = 1 mm. For Masson’s staining: SHAM: *n* = 3; MI + PBS: *n* = 4; MI + 20 mg/kg RHY: *n* = 5; MI + 40 mg/kg RHY: *n* = 5. (**D**) Representative immunoblots showing ANP, BNP, and cTnI expression in serum from mice treated with EX-527 at 4 weeks post-MI. For serum WB analysis: SHAM: *n* = 3; MI + PBS: *n* = 4; MI + RHY: *n* = 3; MI + RHY + EX-527: *n* = 3. Quantification of gray values normalized to internal control, Ponc. S. (**E**) Representative immunoblots showing nuclear FOXO3a and NRF2 levels in the ischemic myocardium of mice treated with EX-527 at 4 weeks post-MI. Quantification of gray values normalized to internal controls, TBP. For cardiac WB analysis: SHAM: *n* = 3; MI + PBS: *n* = 4; MI + RHY: *n* = 3; MI + RHY + EX-527: *n* = 3. Data are presented as mean ± SEM. One-way ANOVA, * *p* < 0.05, ** *p* < 0.01, *** *p* < 0.001, ns, no significance.

In order to verify that the cardioprotective effect of RHY is dependent on SIRT1-targeted activation, we further co-administered a selective SIRT1 inhibitor, selisistat (EX-527) [24], starting from 3 days prior to MI. Echocardiography assessment showed that the protective ability of RHY on cardiac function was completely abolished when co-administered with EX-527 (Figure 5A). Masson’s staining further showed that EX-527 co-administered mice no longer had reduced infarct sizes compared to mice administered with RHY alone (Figure 5B,C). Moreover, co-administration with EX-527 also abolished RHY’s ability to decrease levels of myocardial injury markers ANP, BNP, and cTnI following MI, which reverted to equivalent levels of PBS-administered mice (Figure 5D).

Mechanistic analysis revealed that co-administration of EX-527 completely prevented RHY-induced increase in nuclear levels of SIRT1 downstream transcription factors FOXO3a and NRF2, which were normalized to the levels of the MI + PBS group (Figure 5E). We also performed a small pilot study to exclude the effect that EX-527 itself aggravates myocardial injury. The results showed that EX-527 itself exhibited no deleterious effects on cardiac function and serum levels of ANP, BNP, and cTnI in both sham-operated mice and mice that underwent MI (Supplemental Figure S2); however, this pilot study was carried out for 2 weeks rather than the main study’s endpoint of 4 weeks. Collectively, these results demonstrate that the cardioprotective effect of RHY is dependent upon activation of the SIRT1/NRF2/FOXO3a signaling axis.

### 3.6. RHY Protects Cells from Oxidative Stress via SIRT1 Activation

**Figure 6:**
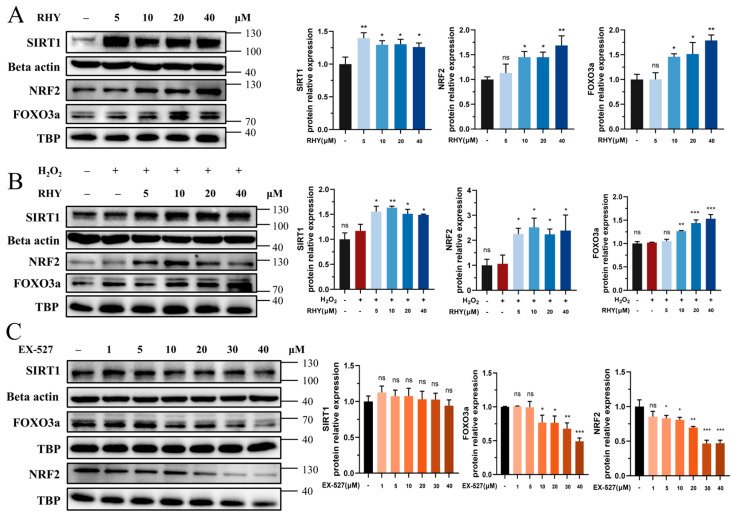
RHY activates the SIRT1/NRF2/FOXO3a signaling axis in vitro. (**A**) Representative immunoblots showing the expression of SIRT1, NRF2 and FOXO3a in H9c2 cells treated with different concentrations of RHY (12h) compared to control. (**B**) Representative immunoblots showing the expression of SIRT1, NRF2 and FOXO3a in H9c2 cells treated with RHY (12h) at different concentrations combined with 200 µM H_2_O_2_ for 1 h. (**C**) Representative immunoblots showing the expression of SIRT1, FOXO3a, and NRF2 in H9c2 cells treated with different concentrations of SIRT1 inhibitor (EX-527). The level of FOXO3a was detected after 12 h, and NRF2 was detected after 24 h. Quantification of gray values normalized to internal control, beta actin for total protein levels and TBP for nuclear protein levels. Data are presented as mean ± SEM. One-way ANOVA, * *p* < 0.05, ** *p* < 0.01, *** *p* < 0.001, ns, no significance. *n* = 3 independent experiments in each group.

To validate the ability of RHY in protecting against oxidative stress via SIRT1-targeted activation, we further examined its effects in vitro using H9c2 cardiomyocytes. RHY at doses of 5–40 µM exhibited no significant decreases in cell viability as determined using CCK-8 assay, as the basis for subsequent experiments (Supplemental Figure S3). Under basal conditions, RHY significantly increased SIRT1 and NRF2/FOXO3a protein levels that were equally evident across all tested concentrations, demonstrating that RHY is a strong SIRT1 activator even in the absence of oxidative stress (Figure 6A). We further treated cells with H_2_O_2_ stimulation to mimic oxidative stress in vitro, which showed a similar trend whereby RHY markedly enhanced the expressions of SIRT1 and NRF2/FOXO3a across all tested concentrations (Figure 6B). These results also suggest that a low concentration of RHY already exhibits strong SIRT1-targeted activation potential and higher doses present no noticeable increases, which corresponds to our in vivo experiment results.

We further examined the ability of RHY in preventing the functional endpoints of oxidative stress. CCK-8 assays showed that RHY treatment protected against H_2_O_2_-induced decrease in cell viability and decreased the levels of H_2_O_2_-induced γ-H2AX, which were abolished by co-treatment with EX-527 (Supplemental Figure S4). Finally, we set out to confirm the functional effect of the SIRT1-specific inhibitor EX-527, which only prevents the functional activity but not protein or mRNA expressions of SIRT1 [25,26]. Indeed, our results showed that EX-527 treatment did not affect total protein levels of SIRT1 but markedly decreased nuclear levels of SIRT1 downstream transcription factors FOXO3a and NRF2, especially at higher dosages (Figure 6C). These in vitro findings further verify that the protective ability of RHY in oxidative stress is via targeted activation of the SIRT1/NRF2/FOXO3a signaling axis.

## 4. Discussion

The major outcome of this study is that RHY administration improves cardiac function post-MI, which is accompanied by improvements in secondary outcomes, including protection against oxidative stress and DNA damage, via activation of the SIRT1/NRF2/FOXO3a signaling axis. One important outcome is the excess generation of oxidative stress, which is widely recognized as a central driver of cardiomyocyte death following myocardial infarction, resulting in adverse patient outcomes, including ventricular remodeling and progression to heart failure [27]. Although current clinical treatment strategies all involve restoring coronary blood flow post-MI, these procedures cannot reverse the damage that has already occurred in the ischemic myocardium because the heart lacks an endogenous repair and regenerative mechanism [28]. Hence, there exists a therapeutic gap for the utilization of various drugs, in particular antioxidants, which can act by limiting the extent of early ischemic damage following MI, although in most cases the efficacy of these drugs is often limited by poor targeting and unfavorable pharmacokinetic properties [29]. In this regard, drugs with antioxidative properties, in particular natural plant alkaloids such as RHY, may present an interesting solution via enhancing the body’s endogenous antioxidant and repair systems while also presenting favorable safety profiles [30,31,32].

A major player involved in intrinsic redox homeostatic pathways is the histone deacetylase SIRT1, which regulates and promotes the functions of various proteins, including transcriptional factors involved in inflammation, apoptosis, oxidative stress, and redox homeostasis [30,31,32]. In the heart, SIRT1 activation is well known for its antioxidative properties in protecting against oxidative stress-induced injury [33]. As such, SIRT1-targeted therapies or drugs that can activate SIRT1 would be beneficial in myocardial infarction, in this case, RHY, which we demonstrated as a robust activator of SIRT1 under basal conditions as well as during oxidative stress. RHY activated the functions of SIRT1 downstream transcription factors NRF2 and FOXO3a, the two key redox-sensitive transcriptional factors critically involved in intracellular oxidative stress and DNA repair responses [34,35]. There also exists a synergistic crosstalk between NRF2/FOXO3a in regulating and amplifying the genes responsible for the cellular antioxidant response [8]. RHY administration not only decreased the levels of intracellular ROS but also decreased markers of oxidative damage, protein tyrosine nitration, and DNA damage. Collectively, our findings suggest that the ability of RHY in activating the SIRT1/NRF2/FOXO3a axis underlines its key role in protecting against MI-induced ischemic injury.

Rhynchophylline has been shown to exhibit beneficial roles in preventing inflammation and protective effects in models of cerebral ischemia and neurodegeneration [14,15,16]. Moreover, because RHY is a key active ingredient of Uncaria rhynchophylla that is commonly used in several traditional Chinese medicine formulations, its antioxidative properties have been previously characterized, including its beneficial effects in cardiac dysfunction [17,18]. Nevertheless, our current study demonstrates for the first time the cardioprotective effects of RHY in a model of myocardial infarction. In particular, we revealed that the ability of RHY in protecting against MI-induced ischemic injury was via targeted activation of SIRT1 and downstream redox-sensitive transcription factors NRF2/FOXO3a, and these effects were abolished upon co-administration of a SIRT1-specific inhibitor.

There are several limitations to our study. First, we only used male C57BL/6 mice for the MI model, which represents only a single strain and sex. Second, all mice were sacrificed at 4 weeks post-MI, hence there was no long-term follow-up. Third, only the permanent coronary artery ligation model was used, and there is a lack of a reperfusion model; hence, our model of MI does not fully represent the clinical reperfusion therapy. Fourth, our study only utilized pharmacological inhibition of SIRT1 via EX-527 rather than global knockdown or knockout models. Fifth, the use of prophylactic RHY administration starting from three days prior to MI until sacrifice does not directly translate to therapeutic treatment of MI, but rather a preconditioning-like cardioprotective effect of RHY. Sixth, our study lacked detailed pharmacokinetic or safety data and therefore, the dose–response pattern of RHY cannot be clearly demonstrated. Seventh, our study mainly focused on the ability of RHY in preventing oxidative stress via activating the SIRT1/NRF2/FOXO3a axis; the preconditioning-like cardioprotective potential of RHY to prevent cardiac remodeling requires further investigation. Eighth, mouse randomization was not done via a computer-generated sequence, but rather stratified by body weight; the allocation was not truly random. Finally, the H9c2 cell line used does not fully reflect adult cardiomyocyte biology and is therefore limited in supporting our in vivo findings.

Nevertheless, our study provides evidence that the robust antioxidative properties of RHY in enhancing the endogenous antioxidant defense capacity of the myocardium are associated with activation of the SIRT1/NRF2/FOXO3a signaling axis. These findings provide a theoretical basis as well as new mechanistic insight into the preconditioning-like cardioprotective effects of RHY during myocardial infarction.

## Data Availability

The original contributions presented in this study are included in the article/Supplementary Material. Further inquiries can be directed to the corresponding authors.

## Supplementary Materials

The following supporting information can be downloaded at: https://www.mdpi.com/article/10.3390/antiox15060669/s1. Supplemental Figure S1: Effect of RHY on Acetylation of FOXO3a and NRF2 in mice following myocardial infarction (MI). Supplemental Figure S2: Effect of EX-527 on cardiac function and protein expression following myocardial infarction (MI). Supplemental Figure S3: CCK-8 assay showing cell viability of H9c2 cells treated with RHY at different concentrations. Supplemental Figure S4: Effect of RHY and EX-527 on cell viability and γ-H2AX expression under H_2_O_2_-induced oxidative stress.

## Abbreviations

The following abbreviations are used in this manuscript:

3-NT	3-nitrotyrosine
ANP	atrial natriuretic peptide
BNP	B-type natriuretic peptide
cTnI	cardiac troponin I
EF	ejection fraction
EX-527	selisistat
FBS	fetal bovine serum
FOXO3A	forkhead box O3A
FS	fractional shortening
γ-H2AX	phosphorylated histone H2AX
H_2_O_2_	hydrogen peroxide
LAD	left anterior descending coronary artery
LV	left ventricular
MI	myocardial infarction
NRF2	nuclear factor erythroid 2–related factor 2
RHY	rhynchophylline
ROS	reactive oxygen species
SIRT1	sirtuin 1
SPF	specific pathogen-free
SV	stroke volume
