# Supplementary material for: Rhynchophylline Protects Against Ischemic Injury Following Myocardial Infarction via Activation of the SIRT1/NRF2/FOXO3a Axis
Source: Antioxidants (Basel). 2026 May 26;15(6):669. doi: 10.3390/antiox15060669 (PMC13295487; doi:10.3390/antiox15060669)
Supplement: Supplementary file 1 [file antioxidants-15-00669-s001.zip › antioxidants-4214763-supplementary.pdf]

Supplemental Figure S1

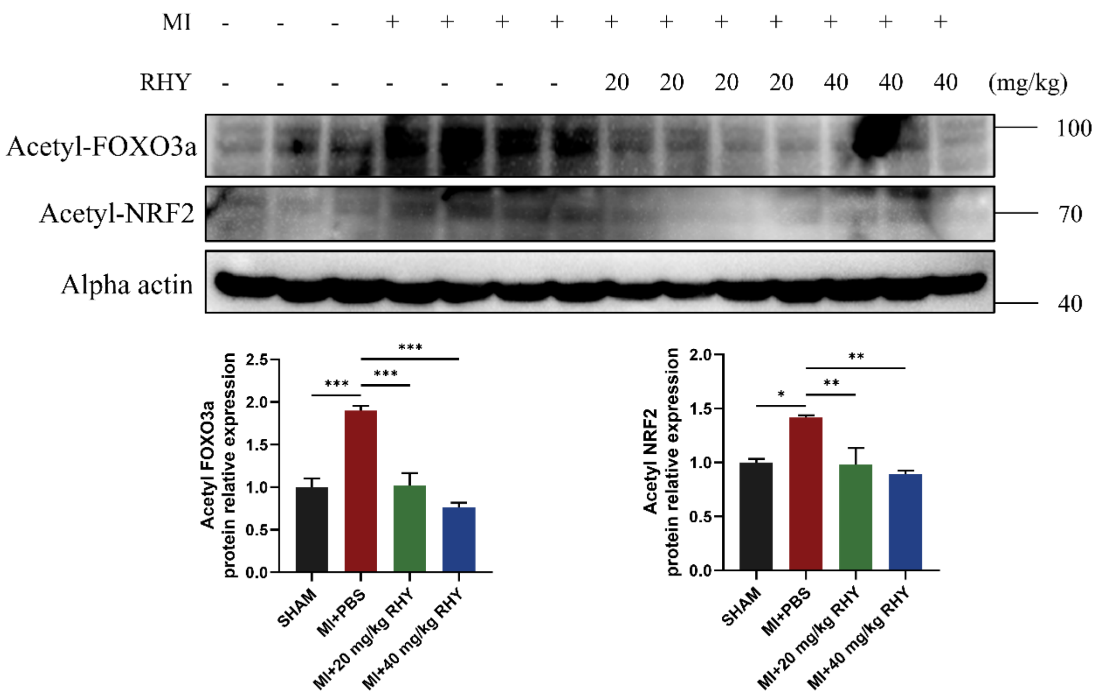

Supplemental Figure S1. Effect of RHY on Acetylation of FOXO3a and NRF2 in mice following myocardial infarction (MI). Representative immunoblots showing the expression of Acetyl-FOXO3a and Acetyl-NRF2 in cardiac tissue from mice treated with PBS or different doses of RHY (20 mg/kg or 40 mg/kg) at 4 weeks post-MI. Quantification of gray values normalized to internal control, alpha actin. SHAM, n = 3, MI+PBS, n = 4, MI+20 mg/kg RHY, n = 4, MI+40 mg/kg RHY, n = 3. Data are presented as mean ± SEM. *$p < 0.05$, **$p < 0.01$, ***$p < 0.001$.

Supplemental Figure S2

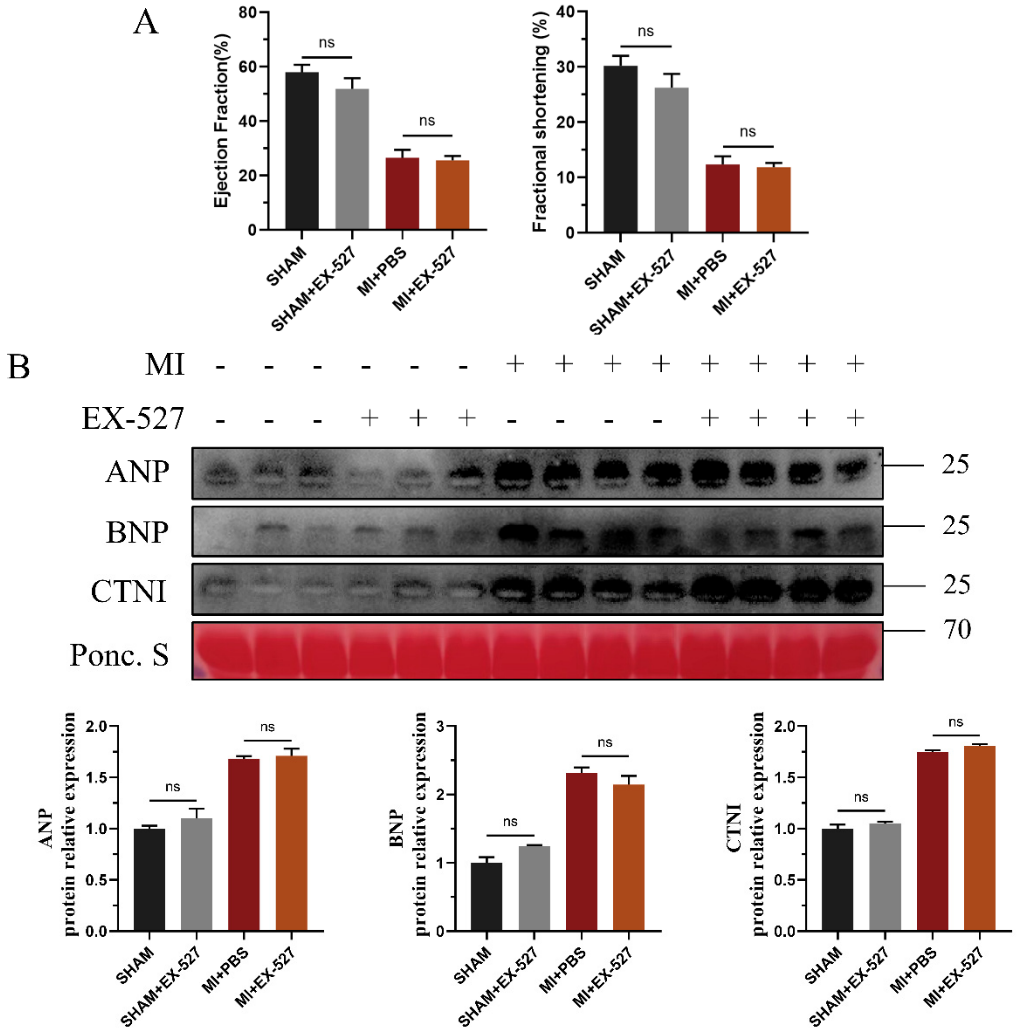

Supplemental Figure S2. Effect of EX-527 on cardiac function and protein expression following myocardial infarction (MI). (A) Cardiac function parameters including ejection fraction (EF%) and fractional shortening (FS%) in mice treated with PBS or EX-527 at 2 weeks post-MI. For echocardiography, SHAM, n = 4, SHAM+EX-527, n = 4, MI+PBS, n = 7, MI+EX-527, n = 7. (B) Representative immunoblot and quantification of Atrial natriuretic peptide (ANP), B-type natriuretic peptide (BNP), and Cardiac troponin I (cTnI) expression in the serum at 2 weeks post-MI from mice treated with EX-527 or PBS. Quantification of gray values normalized to internal control, Ponc. S. For WB analysis, SHAM, n = 3, SHAM+EX-527, n = 3, MI+PBS, n = 4, MI+EX-527, n = 4. Data are presented as mean ± SEM. n.s., no significance.

Supplemental Figure S3

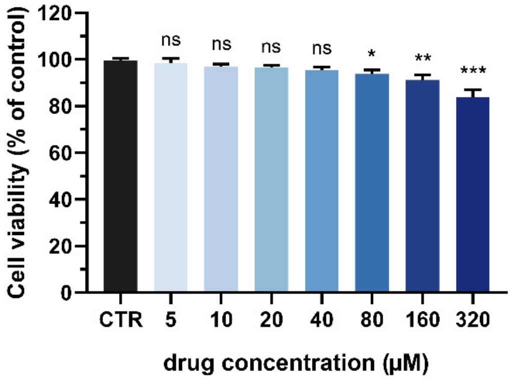

Supplemental Figure S3. CCK-8 assay showing cell viability of H9c2 cells treated with RHY (12h) at different concentrations. n = 18 for each well from 3 independent experiments. Data are presented as mean ± SEM. *$p < 0.05$, **$p < 0.01$, ***$p < 0.001$, n.s., no significance.

Supplemental Figure S4

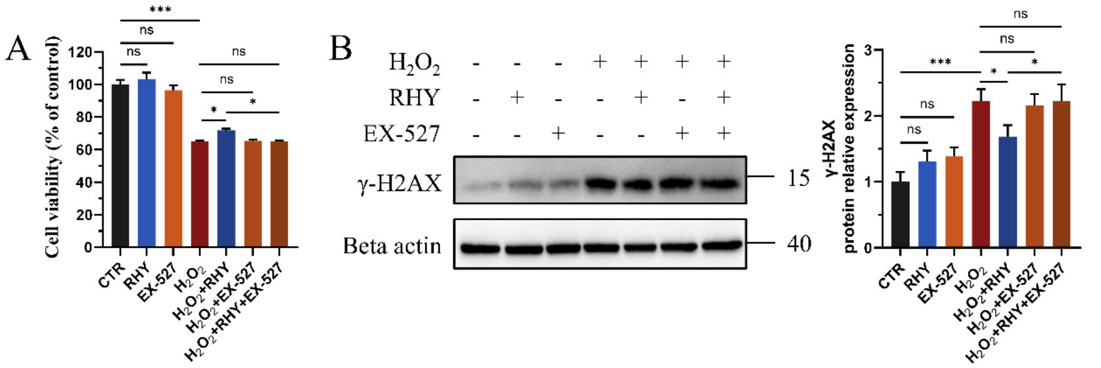

Supplemental Figure S4. Effect of RHY and EX-527 on cell viability and γ-H2AX expression under H_2O_2-induced oxidative stress. (A) Cell viability measured by CCK-8 assay in H9c2 cells treated with RHY (40 μM, 12h), EX-527 (40 μM, 12 h), and/or H_2O_2 (200 μM, 1 h). n = 18 for each well from 3 independent experiments. (B) Representative immunoblot showing γ-H2AX expression in H9c2 cells treated with H_2O_2 (200 μM, 1 h), RHY (40 μM, 12 h), and/or EX-527 (40 μM, 12 h). Quantification of gray values normalized to internal control, alpha actin, n = 3 independent experiments. Data are presented as mean ± SEM. $^*p < 0.05$, $^{**}p < 0.01$, $^{***}p < 0.001$, n.s., no significance.
